# Intestinal Malrotation Associated With Invagination of the Distal Ileum and Cancer of the Cecum: A Case Report and Literature Review

**DOI:** 10.7759/cureus.13637

**Published:** 2021-03-01

**Authors:** Anas Taha, Laura Aniukstyte, Bassey Enodien, Victor Staartjes, Stephanie Taha-Mehlitz

**Affiliations:** 1 Department of Visceral and Thoracic Surgery, Cantonal Hospital Winterthur, Winterthur, CHE; 2 Department of Gastroenterology and Surgery, Vilnius University, Vilnius, LTU; 3 Department of Surgery, Wetzikon Hospital, Wetzikon, CHE; 4 Neurosurgery, Bergman Clinics, Amsterdam, NLD; 5 Clarunis, Department of Visceral Surgery, University Center for Gastrointestinal and Liver Diseases, St. Clara Hospital and University Hospital, Basel, CHE

**Keywords:** intestinal malrotation, midgut malrotation, colon cancer, cecum cancer

## Abstract

Associated midgut malrotation and colon cancer in adult patients is extremely rare and associated with delayed diagnosis and increased morbidity. We present the case of a patient with a three-week history of weakness, diarrhea, and abdominal pain with invagination of the distal ileum. Exploratory laparotomy with ileocecal resection revealed invagination, malrotation, and cecal adenocarcinoma.

## Introduction

Intestinal malrotation is an in-utero developmental disorder, characterized by an improper fixation of the midgut and failure of its normal embryonic rotation between the fifth and 12th week of gestation [1]. This anomaly is estimated to occur in around one in 500-6,000 newborns and presents within the first month of life in 64-80% of the patients [2]. Although most of the cases of intestinal malrotation are recognized as a cause of intestinal obstruction in newborns, its clinical manifestation in adults can be variable and can include nausea, vomiting, dyspepsia, diarrhea, chronic abdominal pain, intestinal obstruction, and volvulus [2-13]. Concomitant midgut malrotation and colon cancer is very rare. We report a case of open hemicolectomy for cecum cancer in a patient with intestinal malrotation and review the literature on this topic.

## Case presentation

**Figure 1:**
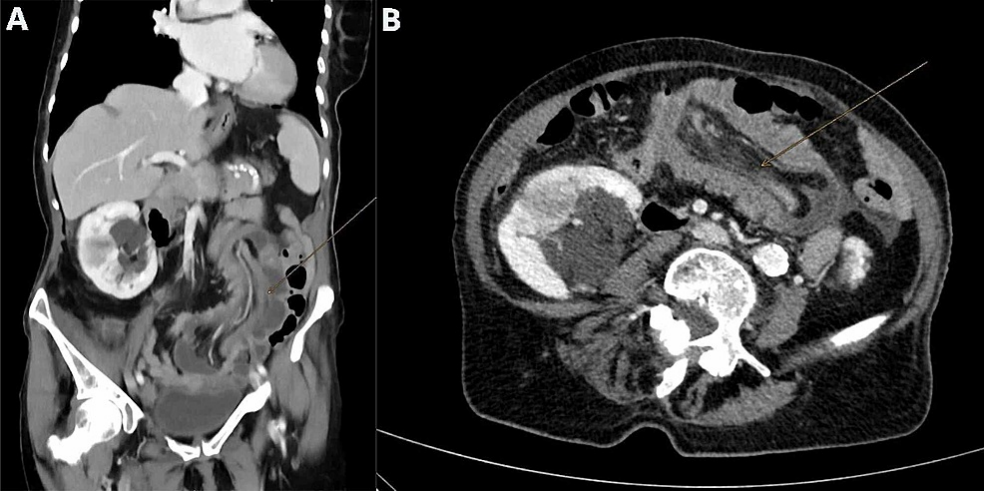
CT image. Pronounced invagination of terminal ileum in the middle abdomen up to the small pelvis with a diameter of 6 cm of invagination of the transverse colon and possible cecal pole in the sigmoid colon and central with mesenteric adipose tissue and peripheral mesenteric vessels and a single lymph node 0.7 cm in diameter and little fluid. Questionable wall thickening in the rectosigmoid transition. Panel A illustrates a coronal and panel B illustrates an axial CT image. CT, computed tomography

An 86-year-old woman with Alzheimer’s disease was admitted to our emergency department after an approximately three-week history of weakness, diarrhea, and abdominal pain. She had a medical history of open appendectomy in adolescence, endoscopic colonic polypectomy, and no previous abdominal pain. Malrotation was not known beforehand. A physical examination revealed conspicuous abdominal tenderness and abdominal distension. No lump was palpable. Laboratory values showed an elevated C-reactive protein (93 mg/L), normal lactate, and anemia (67 g/L) requiring blood transfusion. Abdominal computed tomography (CT) with intravenous contrast (Figure 1) showed a thickened ascending colon with invagination of the distal ileum, as well as imaging features suspicious for sigmoid volvulus. Due to the extensive invagination, an exploratory laparotomy was deemed necessary. Intraoperative examination revealed that the thickened terminal ileum was invaginated into the cecum and reached up to the right colonic flexure. During surgery, we found non-rotation of the intestine: the mobile cecum was located in the midline, but the descending colon was attached to the retroperitoneum, and the small intestine occupied the right side of the abdomen. The distal ileum was carefully and manually released from the colon without any visible serosal lesions. The entry point of invagination appeared to be in the area of the former appendectomy site. Due to the cecal induration at the entry point of the distal ileum, we performed a 7 cm long ileo-cecal resection, with lymph node dissection and ileo-ascending end-to-end anastomosis. Surprisingly, histopathological examination revealed moderately differentiated adenocarcinoma of the cecum (pT4a pN0 (0/14) M0 L0 V0 Pn0 G2), with carcinoma-free resection borders. Postoperatively, the patient was treated with intravenous antibiotics (ciprofloxacin and metronidazole) for 48 hours. We discussed the recommendation for oncological resection (right hemicolectomy) and adjuvant chemotherapy with the patient’s family. Due to the patient’s age, comorbidities, and general condition, the patient underwent no further surgery or chemotherapy. No adverse events or surgical complications were observed during the postoperative course, and the patient was discharged on postoperative day eight.

## Discussion

Intestinal malrotation is a congenital abnormality resulting from a less-than-normal 270° anti-clockwise rotation of the midgut along its vascular pedicle during embryologic development. More than 90% of patients with intestinal malrotation present symptomatically within the first year of life, although malrotation can remain asymptomatic even in adults [10,13]. There are three types of malrotation depending on when the normal growth of middle intestine is interrupted: (1) non-rotation (type IA): when the first 90° anti-clockwise rotation occurs; (2) incomplete intestinal rotation (type II): several abnormalities due to variations in anti-clockwise rotation of the last 180° of the intestine; (3) inverse rotation (type III): when the postarterial section of the midgut first re-enters the abdominal cavity. Non-rotation is the most frequent type of reviewed cases and has been confirmed in 10 patients. The present case also showed findings of non-rotation type, which includes left positioned cecum and ascending colon with right-sided duodenojejunal junction. The development of imaging technology has increased the diagnosis rate of intestinal malrotation. Nowadays, CT is one of the most useful diagnostic examination for intestinal malrotation in adults. On the other hand, many cases of malrotation are still incidentally revealed by operation for concomitant disease, and as in our case, as well as most of the case reports which we reviewed, the intestinal malrotation was discovered only when laparoscopy or laparotomy was carried out. In our literature review, we identified 13 other cases of intestinal malrotation and colon cancer. However, most of the patients identified in our review (Table 1) had symptoms such as chronic abdominal pain, diarrhea or constipation, fatigue, anemia, among others. Table 1 summarizes the cases of colon cancer in patients with intestinal malrotation that are reported in the English literature [2-13]. Most of the case reports indicate cecum cancer (46.2%); ascending colon cancer (30.8%); and transverse colon, descending colon, and sigmoid colon cancer (7.7%) each. The tumor location is mostly diagnosed by colonoscopy. Although there is no direct evidence for the connection between a congenital anomaly and carcinogenesis, Ren and Lu [4] described that intestinal malrotation may cause chronic bowel obstruction resulting in inflammation and colon cancer development. With respect to the surgical approach, in our reviewed reports, open surgery was performed in 53.8% of the cases, laparoscopy in 38.5% of the cases, and one patient (7.7%) underwent conversion to open laparotomy. Laparoscopic surgery for colon cancer is becoming more popular and advanced. On the other hand, intestinal malrotation is associated with vascular abnormality, and it is difficult to safely perform laparoscopic hemicolectomy and lymphadenectomy for patients with this condition. In our case, we preferred laparotomy: first, because of extensive distal ileum invagination into the ascending colon. Second, the presence of ambiguous abdominal CT findings led us to consider laparotomy to be more safe and reliable.

**Table 1:** Literature review for cases of intestinal malrotation and colon cancer. AdenoCa, adenocarcinoma; G1, well-differentiated adenocarcinoma; G2, moderately differentiated adenocarcinoma; CT, computed tomography

Case	Author	Publish	Age	Sex	Presentation	Colon cancer location	Type	Diagnosis of intestinal malrotation	Diagnosis of tumor location	Operation	Histopathology	TNM classification
1.	Brillantino et al. [3]	2009 Italy	34	M	Diarrhea, abdominal pain, weight loss	Cecum	Non-rotation	Operation	Colonoscopy	Open right hemicolectomy, lymphonodectomy	AdenoCa/G1	Unknown
2.	Ren and Yu [4]	2009 China	45	M	Abdominal pain, changed bowel habits	Ascending	Non-rotation	Operation	Operation	Open right hemicolectomy, lymphonodectomy	AdenoCa/G1-G2 + mucinous Ca	Unknown
3.	Michalopoulos et al. [5]	2010 Greece	76	M	Fatigue, constipation, anemia	Ascending	Reversed rotation	Operation	Colonoscopy	Open right hemicolectomy, lymphonodectomy	AdenoCa	Unknown
4.	Morimoto et al. [6]	2012 Japan	57	M	Positive fecal occult blood test	Cecum	Reversed rotation	Operation	Colonoscopy	Laparoscopic ileocecal resection, lymphonodectomy	AdenoCa	pT2N0M0
5.	Donaire et al. [7]	2013 USA	52	M	Lethargy, weight loss, anemia	Ascending	Non-rotation	Operation	Colonoscopy	Laparoscopic >> open right hemicolectomy, lymphonodectomy	Tubular adenoCa	Unknown
6.	Hirano et al. [8]	2013 Japan	82	F	Positive fecal occult blood test	Transverse	Reversed rotation	Contrast enema	Colonoscopy	Laparoscopic transverse colectomy, lymphonodectomy	Tubular adenoCa/G1	Unknown
7.	Hirano et al. [9]	2013 Japan	68	F	Bloody stools	Ascending	Non-rotation	Contrast enema	Colonoscopy	Laparoscopic right hemicolectomy, lymphonodectomy	Tubular adenoCa/G1	Unknown
8.	Norris et al. [2]	2014 UK	64	F	Malaise, fatigue, abdominal pain	Cecum	Non-rotation	Operation	CT scan	Open right hemicolectomy, lymphonodectomy	Unknown	Unknown
9.	Ray and Morimoto [10]	2015 India	60	F	Anemia, abdominal pain, dyspepsia, constipation	Cecum	Non-rotation	Operation	Operation	Open right hemicolectomy, lymphonodectomy	Tubular adenoCa/G2	pT3N1Mx
10.	Nakayama et al. [11]	2016 Japan	63	M	Abdominal pain, abdominal distension, constipation	Descending	Non-rotation	Operation	CT scan	Open left hemicolectomy, lymphonodectomy, appendectomy	Tubular adenoCa/G2	Unknown
11.	Nishida et al. [12]	2017 Japan	53	M	Abdominal discomfort	Sigmoid	Non-rotation	Operation	Colonoscopy	Laparoscopic sigmoid resection, lymphonodectomy	Tubular adenoCa/G1	Unknown
12.	Nakatani et al. [13]	2017 Japan	78	M	Constipation	Cecum	Non-rotation	Operation	Operation	Laparoscopic ileocecal resection, lymphonodectomy	Tubular adenoCa/G1	pT3N0M0
13.	Nakatani et al. [13]	2017 Japan	81	M	Positive fecal occult blood test	Cecum	Non-rotation	Operation	Colonoscopy	Laparoscopic ileocecal resection, lymphonodectomy	Tubular adenoCa/G1	pT3N0M0

## Conclusions

We report the case of a patient with cecum cancer who also had intestinal malrotation and distal ileum invagination. Open surgical resection and lymphadenectomy were performed. Anatomical malrotations can lead to challenging approaches for surgical resection. For safety, surgeons should always consider a laparotomy if malrotation is suspected on imaging beforehand. The causal relationship between intestinal malformation and carcinogenesis is not clearly established and remains to be further investigated.
